# Blunted ventral striatal reactivity to social reward is associated with more severe motivation and pleasure de**fi**cits in psychosis

**DOI:** 10.21203/rs.3.rs-4468839/v1

**Published:** 2024-06-12

**Authors:** Jack Blanchard, Alexander Shackman, Jason Smith, Ryan Orth, Christina Savage, Paige Didier, Julie McCarthy, Melanie Bennett

**Affiliations:** University of Maryland, College Park; University of Maryland, College Park

**Keywords:** anhedonia/avolition, fMRI, incentive delay paradigm, negative symptoms, psychosis/psychotic spectrum, schizophrenia

## Abstract

Among individuals living with psychotic disorders, social impairment is common, debilitating, and challenging to treat. While the roots of this impairment are undoubtedly complex, converging lines of evidence suggest that social motivation and pleasure (MAP) deficits play a key role. Yet most neuroimaging studies have focused on monetary rewards, precluding decisive inferences. Here we leveraged parallel social and monetary incentive delay fMRI paradigms to test whether blunted reactivity to social incentives in the ventral striatum–a key component of the distributed neural circuit mediating appetitive motivation and hedonic pleasure–is associated with more severe MAP symptoms in a transdiagnostic sample enriched for psychosis. To maximize ecological validity and translational relevance, we capitalized on naturalistic audiovisual clips of an established social partner expressing positive feedback. Although both paradigms robustly engaged the ventral striatum, only reactivity to social incentives was associated with clinician-rated MAP deficits. This association remained significant when controlling for other symptoms, binary diagnostic status, or ventral striatum reactivity to monetary incentives. Follow-up analyses suggested that this association predominantly reflects diminished striatal activation during the receipt of social reward. These observations provide a neurobiologically grounded framework for conceptualizing the social-anhedonia symptoms and social impairments that characterize many individuals living with psychotic disorders and underscore the need to establish targeted intervention strategies.

## Introduction

Among individuals living with schizophrenia and other psychosis spectrum disorders, social impairments are common, often debilitating, and challenging to treat, underscoring the importance of clarifying the underlying neurobiology^1–7^. While the roots of social impairment are undoubtedly complex and multiply determined, converging lines of laboratory and experience-sampling data suggest that motivation and pleasure (MAP) symptoms play a key role^3, 8–17^.

MAP deficits are often conceptualized in terms of blunted reactivity to social rewards, including reduced motivation to seek out and engage in social interactions (amotivation/avolition) and diminished hedonic pleasure when interactions do occur (anhedonia)^3, 12, 18–22^. Recent meta-analyses of the psychosis neuroimaging literature provide some neurobiological support for these hypotheses, but modest sample sizes (median n_Cases_=19) and a near-exclusive focus on monetary reward and other non-social incentives precludes decisive inferences^21, 23^

To date, only a handful of psychosis studies have examined potential alterations in neural reactivity to social rewards. Two modest case-control studies provide preliminary evidence of aberrant reactivity to social incentives in the ventral striatum (Supplementary Figure S1)–a key hub in the distributed neural system mediating appetitive motivation (‘wanting’) and hedonic pleasure (‘liking’)^24–26^–among individuals with schizophrenia (n_Cases_=27)^27, 28^. Leveraging a much larger, transdiagnostic psychosis sample (n_Cases_=71), Jimenez and colleagues reported that diminished ventral striatum reactivity to social reward is associated with more severe MAP deficits and social-anhedonia symptoms^29^. Whether this association is reproducible remains unknown and whether it is specific to social reward remains unclear, as the authors only examined social incentives.

**Figure 1:**
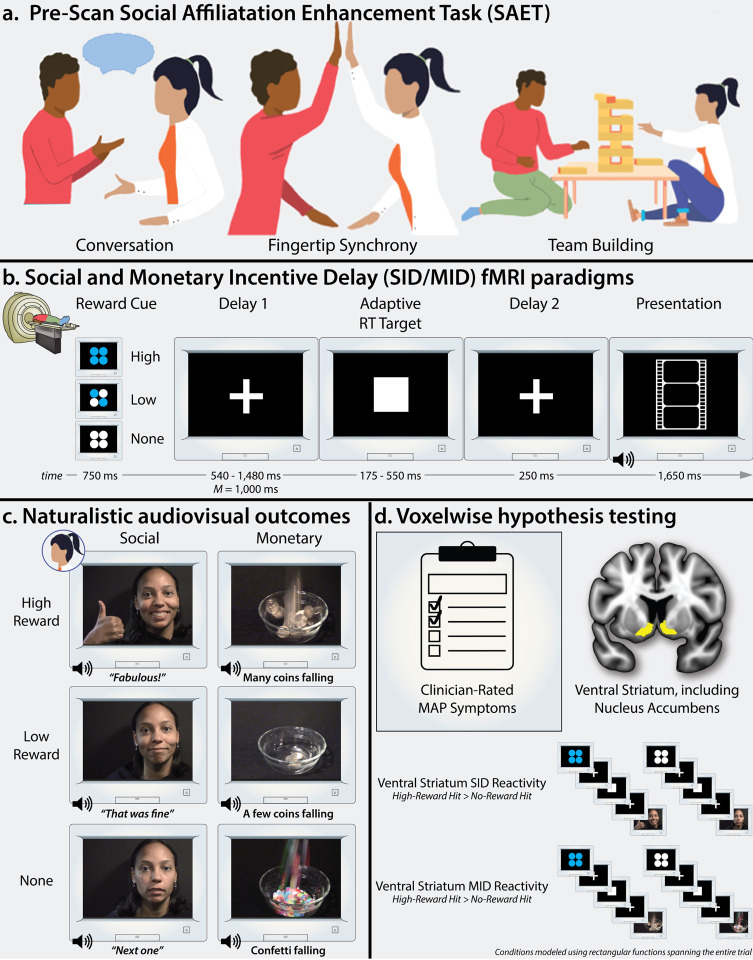
Conceptual overview of the study. **(a) Pre-Scan Social Affiliation Enhancement Task (SAET).** In the first phase of the session, participants completed the SAET, which encompasses 3 tasks–conversation, implicit fingertip synchrony, and team building–aimed at promoting a sense of affiliation with an experimental partner. Prior work confirms the validity of this approach in psychotic samples^14^(**b**) **Social and Monetary Incentive Delay (SID/MID) fMRI paradigms. (c) Naturalistic audiovisual outcomes.** To maximize ecological validity and translational relevance, short audiovisual clips depicting varying degrees of social or monetary reward served as the outcomes. For the SID paradigm, the clips depicted the individual who served as the experimental partner during the SAET. **(d) Voxelwise hypothesis testing**. Hypothesis testing focused on the association between clinician-rated MAP symptoms and ventral striatum *(yellow)* reactivity to social and monetary reward, indexed by the cardinal High-Reward vs. No-Reward contrast. Analyses focused on trials where the participant responded sufficiently fast to earn reward (‘hit’). For primary hypothesis testing, each condition was modeled using a rectangular function spanning the entire trial. **Abbreviations**–fMRI, functional magnetic resonance imaging; MAP, motivation and pleasure; MID, monetary incentive delay paradigm; ms, milliseconds; RT, response time; SID, social incentive delay paradigm.

Here we used fMRI and parallel social and monetary incentive delay (SID/MID) paradigms to test the overarching hypothesis that blunted ventral striatum reactivity to social incentives will be associated with more severe MAP symptoms, indexed using gold-standard clinician ratings (Figure 1). The inclusion of the MID paradigm enabled us to clarify the specificity of this hypothesized association for the first time. Follow-up analyses were used to explore the relevance of other symptom dimensions, narrower reward facets (anticipation vs. presentation), and less intensively scrutinized brain regions. To ensure a broad spectrum of social impairment and MAP symptomatology, we adopted a Research Domain Criteria (RDoC) sampling strategy, focusing on a transdiagnostic community sample that was heavily enriched for psychosis (Table 1)^30, 31^. Most participants were on a stable regimen of outpatient treatment, enhancing clinical relevance. To date, clinical neuroimaging studies of social reward have relied almost exclusively on static photographs of positive facial expressions posed by unfamiliar adult models (i.e., strangers). To maximize ecological validity and translational relevance, we instead capitalized on audiovisual clips of an established social partner expressing varying degrees of social reward. As shown in Figure 1, we used the Social Affiliation Enhancement Task (SAET) to cultivate a sense of affiliation with an experimental partner just before the neuroimaging assessment^10^. Prior work by our group confirms the validity of this approach, demonstrating that the SAET significantly enhances affiliative feelings, perceived closeness, and willingness to interact with the partner^14^. This novel naturalistic approach enabled us to manipulate the intensity of nonverbal (facial expressions and gestures), paralinguistic (vocal intonation), and verbal (praise) indicators of social reward expressed by a social partner.

## Materials / Subjects and Methods

### Study Overview

The present study stems from a larger project focused on the nature and neurobiology of social affiliative deficits in psychosis (R01-MH110462)^14, 32–34^. Participants completed two assessments: a baseline clinical session and a two-phase laboratory session. At the baseline clinical session, eligibility was confirmed; participants provided informed written consent; and demographic, diagnostic, symptom, and other self-report data were acquired. Participants were instructed to abstain from taking sedatives/benzodiazepines for at least 12 hours prior to the MRI assessment. None of these individuals disclosed concerns or exhibited noteworthy withdrawal or rebound effects. Latency between the two sessions was <2 weeks (M=6.5 days, SD=2.9). During the two-phase laboratory session, participants completed (a) the SAET outside the scanner, and (b) the social and monetary incentive delay (SID/MID) paradigms inside the scanner (Figure 1) as well as additional tasks not reported here^14^. Following the last scan, participants were debriefed and compensated. Procedures were approved by the University of Maryland, Baltimore Institutional Review Board.

### Participants

#### Recruitment.

To capture a broad spectrum of motivation and pleasure deficits, maximizing range and statistical power, a mixed transdiagnostic adult sample—including both clinical and community participants—was recruited^31^. A modest number of psychiatrically healthy community participants was included (19.4%; Table 1) to ensure that the full range of affiliative function was captured^31^. Clinical participants were recruited from outpatient community mental health clinics in the Washington, DC-Baltimore metropolitan region. Community participants were recruited via online advertisements (e.g., Craigslist).

#### Enrollment Criteria.

General inclusion criteria included 18–60 years of age, English fluency, and normal or corrected-to-normal vision, and consent to be videotaped during study participation. General exclusion criteria included moderate or severe substance use disorder in the past 6 months or mild substance use disorder in the past month, indexed by the Structured Clinical Interview for DSM-5 Research Version (SCID-5-RV)^35^; standard MRI contraindications; lifetime neurological, developmental, or cognitive disorder, indexed by medical history or cognitive testing; or a lifetime history of serious head injury. Clinical inclusion criteria included a lifetime psychotic disorder (Table 1), clinical stability (i.e., no inpatient hospitalizations in the past 3 months and no changes in psychoactive medication in the past month), indexed by medical history. Community inclusion criteria included absence of current psychiatric diagnoses or medication (past 6 months), and absence of lifetime psychotic, mood, or personality disorder, indexed by SCID-5 and self-report.

#### Final Sample.

A total of 120 participants completed the baseline clinical assessment. Of these, 12 did not attend the neuroimaging session due to psychiatric hospitalization (n=1), study withdrawal (n=10), or inclement weather (n=1). The remaining 108 participants included a mixture of clinical (n=87) and community (n=21) participants. Of these, 39 participants were excluded from analyses due to study withdrawal (n=8), MRI safety concerns (n=5), poor fit in the scanner (n=3), technical problems (n=8), excessive movement (n=1), or inadequate task compliance (hit-rate <10% during any scan; n=14), yielding a final sample of 69 individuals (Table 1). Examining those who are included in imaging analyses (n=69) and those who were excluded (n=39) indicated no significant differences in gender, age, or education (all ps > .05) and no symptom differences as measured by the BPRS and CAINS scales (all ps > .05).

### Clinical Assessments

#### Diagnoses.

Diagnoses were determined using the SCID-5. Assessments were conducted by well-trained Master’s level interviewers supervised by doctoral-level clinical psychologists.

#### Clinician-Rated Symptoms.

The Clinical Assessment Interview for Negative Symptoms (CAINS; Horan et al., 2011; Kring et al., 2013) is a well-established 13-item interview that indexes deficits in Motivation and Pleasure (MAP; 9 items; e.g., amotivation, asociality, and anhedonia; α=0.80) and Expression (4 items; e.g., affective flattening and alogia; α=0.87) (Supplementary Table S1). The CAINS has been successfully deployed in a variety of clinical and non-clinical populations^9, 14, 27, 29, 36, 37^. For hypothesis testing, MAP served as the primary index of social amotivation and anhedonia. The expanded Brief Psychiatric Rating Scale (BPRS) is a 24-item interview that was used to index Positive Symptoms (8 items; α=0.69), Depression/Anxiety (4 items; α=0.74), and Agitation (6 items; α=0.53)^38, 39^

#### Self-Reported Social Function.

The 7-item Interpersonal Relationships scale from the Specific Levels of Functioning (SLOF) instrument was used to index interpersonal functioning (α=0.89) (Supplementary Table S1)^40, 41^. Consistent with prior studies, in the current sample more severe MAP symptoms were associated with poorer interpersonal functioning (r= −.56, p< .001).

### Social Affiliation Enhancement Task (SAET)

The Social Affiliation Enhancement Task (SAET) encompasses a validated suite of procedures for cultivating social rapport, trust, and affiliation (Figure 1a) (for details, see Refs.^10, 14^). Prior work by our group demonstrates that the SAET significantly enhances affiliative feelings, perceived closeness, and willingness to interact with the partner^14^, consistent with work using similar paradigms^17^.

### Social and Monetary Incentive Delay (SID/MID) fMRI Paradigms

#### Overview and Procedures.

As shown in Figure 1b, paralell incentive-delay paradigms were used to probe neural reactivity to social and monetary reward^42, 43^. Both paradigms took the form of balanced 3-condition (Reward Level: High, Low, None) randomized, event-related, repeated-measures designs (paradigm order counterbalanced; 2 scans/paradigm; 22 trials/condition/scan). General task structure, timing, and procedures were identical across paradigms. Because we did not harbor a strong a priori hypothesis about the impact of MAP symptoms on the anticipation-versus-presentation of social reward, trial timing was optimized via simulations to maximize the detection of global differences in reward sensitivity, while remaining mindful of participant burden and tolerability (variance inflation factors < 2.55). Participants were completely informed about the task structure and contingencies prior to scanning. They were instructed that the goal of both paradigms was to maximize reward receipt and that this was contingent on the speed of their response to a briefly presented visual target. Responses were made using the first digit of the dominant hand and an MRI-compatible response-pad (MRA, Washington, PA). To maintain a comparable level of difficulty across paradigms, trials, and participants, the response-time threshold (signaled by the duration of the target presentation) was adaptively adjusted on a trial-by-trial basis (±25-ms; target hit-rate: 66%). Too-slow responses (‘misses’) triggered the presentation of the No-Reward audiovisual clips (Figure 1c). No-Reward clips were presented on all No-Reward trials, irrespective of response time (hit/miss). Prior to scanning, participants practiced abbreviated versions of the paradigms and staff provided feedback as necessary to ensure participant comprehension. Stimulus presentation and behavioral data acquisition was controlled using Presentation (version 19.0, Neurobehavioral Systems, Berkeley, CA). Hit-rate was matched across paradigms, t(68)=0.85, p=0.40 (SID: M=64.8%, SD=0.08; MID: M=65.5%, SD=0.05) and unrelated to the severity of MAP symptoms, |r| <0.08, p>0.51.

#### SID Outcomes.

Prior neuroimaging studies of social reward in psychosis have relied on static photographs of positive facial expressions posed by unfamiliar adult models^27–29^. Here we capitalized on naturalistic audiovisual clips of the experimental partner from the SAET, enhancing ecological validity and translational relevance (Figure 1). Building on preclinical work in university students^44^, this approach enabled us to manipulate the intensity of nonverbal (facial expressions and gestures), paralinguistic (vocal intonation), and verbal (praise) indicators of social reward expressed by a psychologically meaningful social partner. High-Reward clips featured large open-mouth smiles, thumbs-up gestures, and verbal feedback indicative of exceptional performance (Amazing!, Awesome! Fabulous! Fantastic!, Spectacular!) and expressed in an ebullient manner (Figure 1c). Low-Reward clips featured small closed-mouth smiles and verbal feedback indicative of good performance (Decent, That was cool, That was fine, That was nice, That was neat), expressed in a mildly positive manner. No-Reward clips were devoid of facial expressions and gestures; instead, the partner simply instructed the participant to prepare for the next trial (Continue, Get ready, Keep going, Next one, Proceed) in a neutral monotone.

#### MID Outcomes.

As shown in Figure 1c, High-Reward audiovisual clips featured 10 coins falling into a bowl, Low-Reward clips featured 4 coins falling in a bowl, and No-Reward trials featured confetti falling into a bowl. In addition to the audiovisual clips, successful performance of the High- and Low-Reward MID trials was incentivized by $1.00 and $0.20, respectively, in monetary compensation. On average, participants earned $32.59 (SD=1.25).

### MRI Data Acquisition

MRI data were acquired using a Siemens Magnetom TIM Trio 3 Tesla scanner (32-channel head-coil). Foam inserts were used to mitigate potential motion artifact. To further mitigate motion artifact, for the final 14 participants, a strip of medical tape was positioned just above the forehead, providing tactile feedback^45^. Sagittal T1-weighted anatomical images were acquired using a magnetization prepared rapid acquisition gradient echo (MPRAGE) sequence (TR=2,400 ms; TE=2.01 ms; inversion=1,060 ms; flip=8°; slice thickness=0.8 mm; in-plane=0.8 mm^2^; matrix=300×320; field-of-view=240×256). A T2-weighted image was collected co-planar to the T1-weighted image (TR=3,200 ms; TE=564 ms; flip=120°). To enhance resolution, a multi-band sequence was used to collect oblique-axial echo planar imaging (EPI) volumes (acceleration=6; TR=1,250 ms; TE=39.4 ms; flip =36.4°; slice thickness=2.2 mm, number slices=66; in-plane=2.1875 mm^2^; matrix=96×96; 355 volumes × 4 scans). Images were collected in the oblique axial plane (approximately −20° relative to the AC-PC plane) to minimize potential susceptibility artifacts. The scanner automatically discarded 7 volumes prior to the first recorded volume. To enable fieldmap correction, two oblique-axial spin echo (SE) images were collected in each of two opposing phase-encoding directions (rostral-to-caudal/caudal-to-rostral) co-planar to the functional volumes (TR=7,220 ms; TE=73 ms). Respiration and pulse were acquired using a respiration belt and photo-plethysmograph affixed to the first digit of the non-dominant hand. Participants were continuously monitored using an MRI-compatible eye-tracker (Eyelink 1000; SR Research, Ottawa, Ontario, Canada) and the AFNI real-time motion plugin^46^. Eye-tracking data were not recorded.

### MRI Data Processing Pipeline

Methods were optimized to minimize spatial normalization error and other potential sources of noise, and are similar to those detailed in other recent reports by our group^14, 47, 48^. Data were visually inspected before and after processing for quality assurance. All participants provided 4 usable scans.

#### Anatomical Data.

T1-weighted images were inhomogeneity corrected using N4^49^ and filtered using ANTS Denoiselmage^50^. Brains were extracted using BEaST^51^ and brain-extracted-and-normalized reference-brains^52^. Brain-extracted T1 images were normalized to a version of the brain-extracted 1-mm T1-weighted MNI152 (version 6) template modified to remove extracerebral tissue^53^. Normalization was performed using the diffeomorphic approach implemented in SyN (version 2.3.4)^50^. T2-weighted images were rigidly co-registered with the corresponding T1 prior to normalization. The brain-extraction mask from the T1 was then applied. Tissue priors were unwarped to native space using the inverse of the diffeomorphic transformation^54^. Brain-extracted T1 and T2 images were segmented—using native-space priors generated in FAST (version 6.0.4)^55^—for subsequent use in T1-EPI co-registration (see below).

#### Fieldmap Data.

SE images and topup were used to create fieldmaps. Fieldmaps were converted to radians, median-filtered, and smoothed (2-mm). The average of the motion- and distortion-corrected SE images was inhomogeneity corrected using N4 and masked to remove extracerebral voxels using 3dSkullStrip (version 20.2.14).

#### Functional Data.

EPI files were de-spiked using 3dDespike, slice-time corrected to TR-center using 3dTshift, and motion corrected to the first volume using ANTS (12-parameter affine). Transformations were saved in ITK-compatible format for subsequent use^56^. The first volume was extracted and inhomogeneity corrected for EPI-T1 co-registration. The reference EPI volume was simultaneously co-registered with the corresponding T1-weighted image in native space and corrected for geometric distortions using boundary-based registration^55^. This step incorporated the previously created fieldmap, undistorted SE, T1, white matter (WM) image, and masks. To minimize potential normalization error, reference EPI volumes were spatially normalized to the MNI template using SyN, intensity standardized, and averaged to create a study-specific EPI template^57–59^. Normalized EPI reference volumes were then normalized to the study-specific. To minimize incidental spatial blurring, the operations necessary to transform each EPI volume from native space to the reference EPI, from the reference EPI to the T1, from the T1 to the MNI template, and from the MNI template to the study-specific EPI template were concatenated and applied to the processed EPI data in a single step. Normalized EPI data were resampled (2 mm^3^) using fifth-order b-splines and spatially smoothed (6-mm) using 3DblurInMask

### fMRI Data Modeling

#### General Approach.

For each participant, first-level modeling was performed using general linear models (GLMs) implemented in SPMT2 (version 7771), using the default autoregressive model and temporal band-pass filter set to the hemodynamic response function (HRF) and 128 s^60^. Consistent with past work^14, 47, 48^, nuisance variates included volume-to-volume displacement and its derivative, motion (6 standard parameters, global volume-to-volume displacement, and temporal derivatives), cerebrospinal fluid (CSF) signal, instantaneous pulse and respiration signals, and ICA-derived nuisance signals (e.g., global motion)^61^. Volumes with excessive volume-to-volume displacement (>0.66 mm) were censored. The inter-trial interval served as the implicit baseline.

#### Hypothesis Testing.

For hypothesis testing purposes, reward signals were modeled using variable-duration rectangular (‘box-car’) regressors that spanned the entire trial, separately for each combination of reward level (High, Low, None) and outcome (Hit/Miss) (Figure 1b). Regressors were convolved with a canonical hemodynamic response function (HRF) and its temporal derivative.

#### Reward Anticipation and Presentation.

To explore the relevance of finer differences in neural reward signaling, we separately modeled the anticipation and presentation phases of the trial using delta functions time-locked to the onset of the cue and outcome, respectively, for each combination of reward level and outcome (Figure 1b). Although our incentive-delay paradigms were not originally optimized for this modeling approach, collinearity proved acceptable (variance inflation factors <3.36)^62^. Regressors were convolved with a canonical HRF.

### Analytic Strategy

#### Overview.

Analyses were implemented in SPSS (version 27.0.1; IBM, Armonk, NY), SPM12^60^, and in-house MATLAB code (version 9.14.0.2239454; The MathWorks, Natick, MA). Diagnostic procedures and data visualizations were used to confirm that test assumptions were satisfied^63^ and key conclusions remained unchanged using robust regression (not reported)^64^. Some figures were created using created using R (version 4.0.2)^65^, Rstudio (version 1.2.1335)^66^, ggplot2 (version 3.4.1)^67^, and MRIcron (version 1.0.20190902)^68^. Clusters and peaks were labeled using the Harvard–Oxford atlas^69–71^, supplemented by descriptions of the orbitofrontal cortex, the ventral striatum, and its two major divisions: the core and shell (Supplementary Figure S1)^72–77^.

#### Confirmatory Testing.

Whole-brain voxelwise (‘second-level’) repeated-measures (‘random effects’) general linear models (GLMs) were used to confirm that the SID and MID tasks robustly engaged the ventral striatum, as indexed by the cardinal High-versus-No-Reward contrast for hit trials. Significance was assessed using p<0.05, whole-brain familywise error (FWE) corrected for cluster extent, and a cluster-defining threshold of p<0.001^78^.

#### Hypothesis Testing.

The overarching goal of this study was to test the hypothesis that blunted ventral striatum reactivity to social incentives is associated with more severe clinician-rated MAP symptoms. To do so, we used a standard voxelwise regression, with mean-centered CAINS MAP as the predictor, mean-centered biological sex and age as nuisance variates, and the High-versus-No-Reward contrast as the outcome (Figure 1d), consistent with prior work^26, 28^. Significance was assessed using p<0.05, FWE corrected for the volume of the anatomically defined ventral striatum (Figure 1d)^79^. The same approach was used to probe potential associations with ventral striatum reactivity to monetary incentives.

#### Specificity Analyses.

When a significant association was observed, a voxelwise multiple regression was used to test whether ventral striatum reactivity to that incentive (e.g., social) continued to explain significant variance in MAP symptoms when statistically controlling for mean-centered reactivity to the other incentive (e.g., monetary), sex, and age (p<0.05, ventral striatum FWE corrected). For a similar voxelwise-covariate approach, see Ref.^80^. Follow-up analyses also allowed us to test whether MAP symptoms explain significant variance in ventral striatum reward signaling, over-and-above mean-centered affective flattening/alogia, positive symptoms, depression/anxiety, agitation, and binary diagnostic status (i.e., case-versus-control; p<0.05, ventral striatum FWE corrected).

#### Secondary Analyses.

The same general approach was used to determine the relevance of disaggregating striatal responses to the anticipation-versus-presentation of reward (see above for fMRI modeling details). Here again, when a significant association was detected, voxelwise multiple regression was used to test whether ventral striatum reactivity to that phase of the trial (e.g., anticipation) continued to explain significant variance in MAP symptoms when statistically controlling for mean-centered reactivity to the other phase (e.g., presentation), sex, and age (p<0.05, ventral striatum FWE corrected). For a similar approach, see Ref.^81^.

#### Exploratory Analyses.

Voxelwise regressions were used to explore potential associations between ventral striatum reward signaling and self-reported interpersonal functioning (SLOF, p<0.05, ventral striatum FWE corrected), and to assess associations between MAP symptoms and reward signaling in other, less intensively scrutinized brain regions (p<0.05, whole-brain FWE corrected).

## Results

### Social and Monetary Incentives Robustly Engage the Ventral Striatum

**Figure 2:**
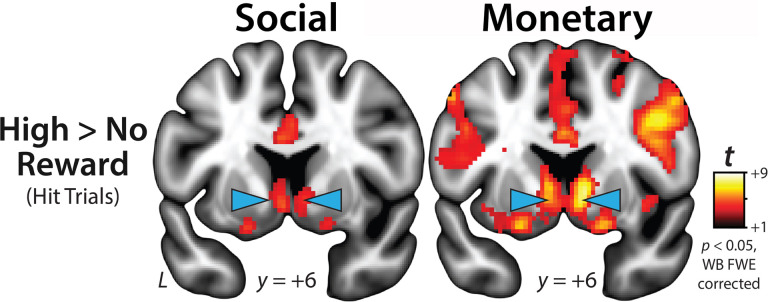
Social and monetary incentives robustly engage the ventral striatum. Figure depicts regions showing significantly greater activation during High-Reward compared to No-Reward hit trials for the SID *(left)* and MID *(right)* paradigms (*p*<0.05, whole-brain FWE corrected). Each condition was modeled using a rectangular regressor spanning the entire trial. Blue arrows indicate the ventral striatum. For detailed results, see **Supplementary Tables S2-S5. Abbreviations**–FWE, familywise error; L, left hemisphere; WB, whole-brain.

As a precursor to hypothesis testing, we used whole-brain voxelwise GLMs to determine whether the SID and MID paradigms had the expected neurophysiological consequences, as indexed by the cardinal High Reward vs. No-Reward contrast (hit trials). Consistent with work in healthy^82, 83^ and psychotic^21, 23^ samples, results confirmed that social and monetary incentives recruited an overlapping network of subcortical and cortical regions, including bilateral ventral striatum, thalamus, cingulate (subgenual, pregenual, and midcingulate), anterior insula, orbitofrontal cortex (posterior orbital gyrus), superior parietal lobule, and ventral visual cortex (p<0.05, whole-brain FWE corrected; Figure 2; Supplementary Tables S2-S5).

### Accumbens Reactivity to Social Incentives is Uniquely Associated with MAP Deficits

**Figure 3:**
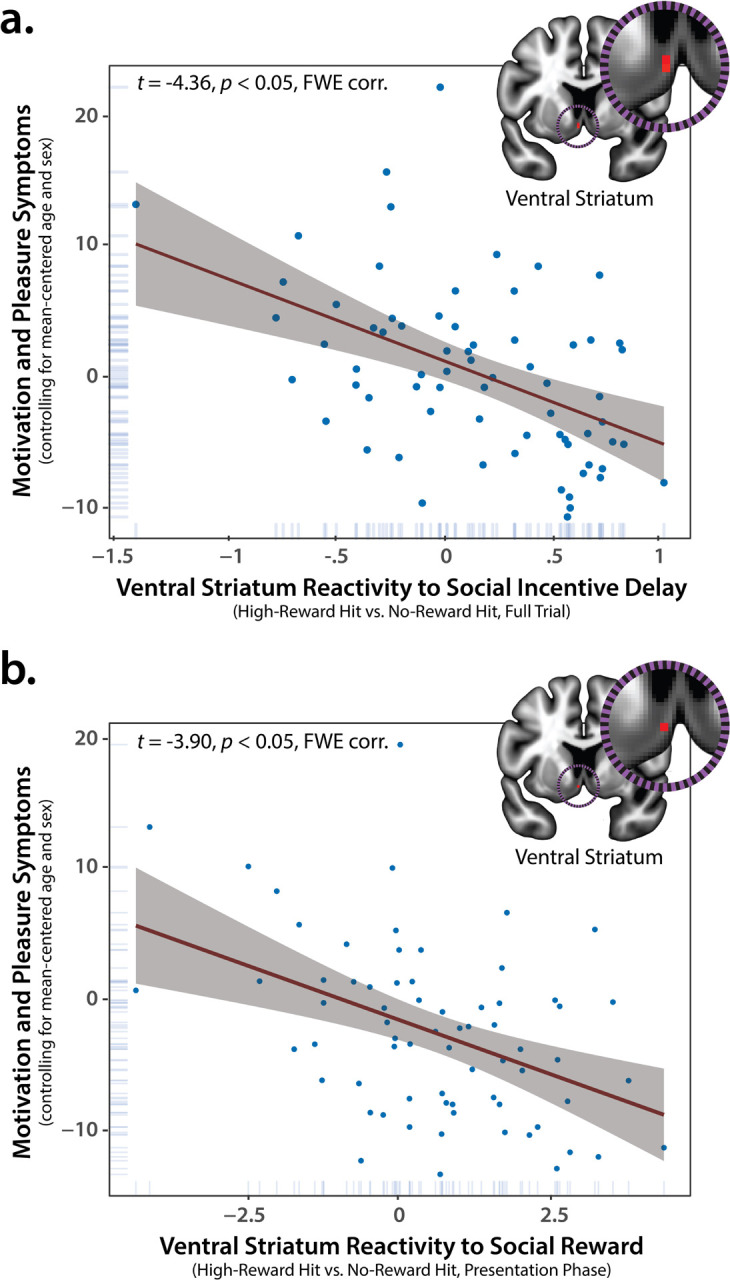
Blunted ventral striatum reactivity to naturalistic social incentives is associated with more severe clinician-rated MAP deficits. **(a)**Decreased ventral striatum activation during High-Reward SID trials *(red)* is associated with more severe MAP symptoms. The cluster peak lies in the putative region of the medial shell of the nucleus accumbens (NACs; x= −4, y= 6, z= −8; cf. **Supplementary Figure S1**). (**b**) Decreased activation during the presentation phase of High-Reward SID trials in an overlapping region of the medial NACs *(red)* is associated with more severe MAP symptoms (x = −4, *y=* 6, z= −8). Red lines depict the regression slope for the peak voxel. Gray envelopes depict 95% confidence intervals. Blue dots and ticks indicate individual participants. Analyses controlled for mean-centered age and biological sex (*p*< 0.05 FWE corrected for the volume of the anatomically defined ventral striatum). Key conclusions remained unchanged for analyses employing robust regression. **Abbreviations–corr**., corrected for the volume of the anatomically defined ventral striatum; FWE, familywise error; MAP, motivation-and-pleasure symptoms (CAINS); SID, social incentive delay paradigm.

We used a standard voxelwise regression to test whether ventral striatum reactivity to social incentives–indexed by the High-versus-No-Reward contrast (hit trials)—is associated with MAP symptoms (Figure 1). As shown in Figure 3a, results revealed a significant cluster in the left ventral striatum where this pattern was evident (p<0.05, FWE corrected for the volume of the ventral striatum; controlling for mean-centered age and sex), with the peak lying in the putative region of the medial shell of the nucleus accumbens (NACs; cf. Supplementary Figure S1). Leveraging the same analytic approach, no significant associations were evident for ventral striatum reactivity to monetary incentives. Consistent with this observation, a voxelwise multiple regression demonstrated that ventral striatum reactivity to social incentives continued to explain significant variance in MAP symptoms while statistically controlling for mean-centered reactivity to monetary incentives (p<0.05, ventral striatum FWE corrected). The association between ventral striatum reactivity to social incentives and MAPS symptoms also remained significant when individually controlling for other symptoms (affective flattening, positive symptoms, depression/anxiety, agitation) or binary diagnostic status (i.e., case-versus-control; p<0.05, ventral striatum FWE corrected). Taken together, these findings demonstrate that blunted ventral striatum reactivity to naturalistic social incentives (Figure 1) is uniquely associated with the severity of clinician-rated MAP deficits.

### Accumbens Reactivity to the Presentation of Social Rewards is Uniquely Associated with MAP Deficits

Using the same general analytic approach, secondary analyses enabled us to examine the potential relevance of disaggregating ventral striatum responses to the anticipation-versus-presentation of social reward (Figure 1). We began by using a whole-brain voxelwise GLMs to determine whether the two phases of the SID paradigm, here considered separately, recruit the ventral striatum. As shown in Supplementary Figure S2, significant ventral striatum activation was only evident during the presentation of social rewards (p<0.05, whole-brain FWE corrected; Supplementary Tables S6-S7). The same pattern was evident using a more liberal small-volume threshold (p<0.05, ventral striatum FWE corrected). Next, we used a voxelwise regression to determine whether ventral striatum activation during the presentation of social rewards is associated with more severe MAP symptoms. As shown in Figure 3b, this pattern was again evident in the medial NACs, overlapping the ventral striatum cluster identified in our primary analyses (p<0.05, ventral striatum FWE corrected; Figure 3a). Ventral striatum activation during the presentation phase continued to explain significant variance in MAP symptoms when statistically controlling for mean-centered activation during the anticipation phase, sex, and age (p<0.05, ventral striatum FWE corrected). In short, diminished ventral striatum reactivity to the receipt of naturalistic social rewards is uniquely associated with the severity of MAP deficits.

### Exploratory Analyses

Ventral striatum reactivity to social and monetary incentives was unrelated to variation in self-reported social functioning (p<0.05, ventral striatum FWE corrected). Whole-brain voxelwise analyses did not detect any significant associations between MAP symptoms and reactivity to either social or monetary incentives outside of the ventral striatum (p<0.05, whole-brain FWE corrected).

## Discussion

The present results demonstrate that blunted ventral striatum reactivity to naturalistic social incentives (Figure 1) is associated with more severe clinician-rated MAP symptoms (Figure 3a). This association remained significant when controlling for a variety of other symptoms (e.g., diminished expressivity) or for diagnostic status, underscoring the utility of conceptual models–such as RDoC and the Hierarchical Taxonomy of Psychopathology (HiTOP)–centered on transdiagnostic symptom dimensions^30, 31, 84, 85^ Although the ventral striatum was robustly engaged by both social and monetary incentives (Figure 2), reactivity to monetary incentives was unrelated to MAP deficits. Consistent with this nil result, in a simultaneous regression model, ventral striatum reactivity to social incentives was uniquely and significantly associated with dimensional variation in MAP symptoms, over-and-above that accounted for by monetary incentives. Secondary analyses demonstrated that diminished reactivity to the presentation of naturalistic social rewards in an overlapping region of the ventral striatum was associated with greater MAP deficits (Figure 3b), replicating and extending work focused on conventional social-reward stimuli (photographs of smiling faces)^29^. This association remained significant when controlling for activation during the earlier reward-anticipation phase, suggesting a unique link between striatal reactivity to positive social feedback and MAP symptoms. Taken together, these observations provide a novel neurobiologically grounded framework for conceptualizing the social deficits that characterize many individuals living with psychotic disorders.

Clinical neuroscientists have long suspected that alterations in ventral striatum function might contribute to the pathophysiology of psychosis, but the specific mapping from brain to symptomatology has only recently begun to come into focus^29, 86, 87^. The present results indicate that more severe MAP symptoms are preferentially associated with blunted reactivity to the receipt of social reward in the medial NACS, a region thought to play a mechanistically critical role in opioid/cannabinoid-mediated hedonic pleasure (‘liking’ reward)^24, 25^ (Supplementary Figure S1). For example, preclinical neuroimaging research shows that acute administration of the opioid antagonist naloxone dampens both subjective pleasure and medial NACs reactivity to positive social stimuli (erotic photographs)^88^. Paralleling our results, dampening was weak-to-nonexistent for monetary stimuli or for the anticipation of social stimuli. Taken together, these observations motivate the hypothesis that, among individuals living with psychosis, more severe MAP deficits reflect aberrant opioid/cannabinoid signaling in the medial NACs during normatively rewarding social interactions, manifesting as diminished feelings of pleasure. While the molecular neurobiology remains untested, prior work by our group supports the psychological component of this hypothesis, showing that individuals with more severe MAP symptoms experienced lower levels of positive affect and social affiliation and emitted fewer positive facial expressions during interactions with a social partner in the SAET, the same individual who served as the model for our naturalistic social-reward stimuli (Figure 1)^14^. Outside of the laboratory, ecological momentary assessment research shows that more severe MAP symptoms are associated with less time spent with others and diminished positive affect in unstructured social contexts^15^. A key challenge for the future will be to clarify the origins and timing of social-reward deficits in psychosis. In particular, it will be fruitful to determine if blunted ventral striatum reactivity to social reward precedes and promotes the emergence of frank psychosis or whether it reflects a consequence of the social isolation and rejection often experienced by individuals with psychotic disorders^2, 89–91^.

The current study elaborates on our prior results^14^ from the parent research program that explored affiliative deficits in psychosis spectrum disorders. Specifically, in that prior study we found that in response to cues of threat, motivation and pleasure deficits undermine the neuroregulatory benefits of social affiliation.^14^ Together with the current findings, these studies indicate that more severe motivation and pleasure symptoms are broadly related to diminished neural responses typically associated with affiliation and social reward. Furthermore, our findings across these two studies suggest that in the social sphere pleasure deficits in psychosis spectrum disorders can occur in the consummatory phase and are not limited to the anticipatory phase^92, 93^.

Clearly, important challenges remain. First, our study was focused on a transdiagnostic community sample enriched for stable psychosis (Table 1). Moving forward it will be important to expand this to more nationally representative samples, and to explore finer-grained differences across diagnostic syndromes (e.g., Schizophrenia vs. Bipolar Disorder) and between clinical and community participants. Second, most participants were on a stable regimen of outpatient treatment. While this approach enhances clinical relevance, the impact on our findings is unknown. Since medication types and dosages were clinically determined, and as we are lacking information on actual medication adherence, we are not able to determine what impact if any medication may have. Third, while our results highlight the importance of the medial NACs, MAP deficits are complex, multi-dimensional, and likely reflect multiple distributed networks. It will be important to understand how interactions between the ventral striatum and other regions implicated in appetitive motivation and hedonic pleasure support variation in MAPS symptoms. Fourth, the absence of punishment trials precludes strong claims about valence^94^. While unlikely, similar associations might be evident for negative social feedback. Finally, given findings that self-assessments of social functioning (as used in the current study) may show discrepancy with informant ratings^95,96^ it will be informative to further examine neural associations with functioning by broadening functional assessments to include high-contact informants and ecological momentary assessment^97^

In sum, the present study leveraged matched SID/MID fMRI paradigms and naturalistic social rewards to demonstrate that MAP symptoms are uniquely associated with blunted ventral striatum reactivity during the receipt of social reward in psychosis. These observations provide fresh neurobiological insights into the social-anhedonia symptoms and social impairment that afflict many individuals with psychotic disorders^1–6, 98, 99^. While established treatments often fail to alleviate these symptoms–making this a critical unmet need^7, 87, 100–102^-our results underscore the potential benefit of emerging interventions targeting positive affect, hedonic pleasure, and social affiliation^17, 101–105^. From an experimental therapeutics perspective^100, 101^, it will be helpful to determine whether such interventions normalize NACs reactivity to social reward.

## Figures and Tables

**Table 1.** Sample Characteristics

Characteristic	Mean (*SD*) or *n* (%)
Age (years)	43.38 (12.02)
Sex	
Male	45 (65.2%)
Female	24 (34.8%)
Race	
African American	46 (66.7%)
White	16 (23.2%)
Asian	3 (4.3%)
More than one race	3(4.3%)
Not Reported	1 (1.4%)
Ethnicity	
Non-Hispanic or Latino	65 (94.2%)
Hispanic or Latino	3 (4.3%)
Not Reported	1 (1.4%)
Education (years)	13.04 (2.41)
Current Employment	
Yes	25 (36.2%)
No	44 (63.8%)
Diagnosis	
Schizophrenia	22 (31.88%)
Schizoaffective, Bipolar Type	6 (8.70%)
Schizoaffective, Depressive Type	9 (13.04%)
Bipolar I with psychotic features	8 (11.59%)
MDD with psychotic features	6 (8.70%)
No diagnosis	18 (26.09%)
Antipsychotic Medication	
Typical	33 (47.82%)
Atypical	6 (8.70%)
Both	4 (5.80%)
Neither	7 (10.14%)
No Medication	19 (27.54%)

Note. *N=69*. **Abbreviations**–MDD, Major Depressive Disorder.

## Data Availability

De-identified raw data are publicly available via the National Institute of Mental Health Data Archive (https://nda.nih.gov/edit_collection.html?id=2480).
